# Prevalence of frailty in Canadians 18–79 years old in the Canadian Health Measures Survey

**DOI:** 10.1186/s12877-017-0423-6

**Published:** 2017-01-21

**Authors:** Dustin Scott Kehler, Thomas Ferguson, Andrew N. Stammers, Clara Bohm, Rakesh C. Arora, Todd A. Duhamel, Navdeep Tangri

**Affiliations:** 1 Health, Leisure & Human Performance Research Institute, Faculty of Kinesiology and Recreation Management, University of Manitoba, 212 Active Living Centre, Winnipeg, MB R3T 2N2 Canada; 2 Institute of Cardiovascular Sciences, St. Boniface Hospital Research Centre, Winnipeg, Canada; 3 Seven Oaks Hospital Research Centre, Winnipeg, Canada; 4 Max Rady College of Medicine, University of Manitoba, Winnipeg, Canada; 5 Department of Surgery (Cardiac surgery), Faculty of Medicine, University of Manitoba, Winnipeg, Canada

**Keywords:** Frailty, Younger age, Coss-Sectional studies, Epidemiology

## Abstract

**Background:**

There is little certainty as to the prevalence of frailty in Canadians in younger adulthood. This study examines and compares the prevalence of frailty in Canadians 18–79 years old using the Accumulation of Deficits and Fried models of frailty.

**Methods:**

The Canadian Health Measures Study data were used to estimate the prevalence of frailty in adults 18–79 years old. A 23-item Frailty Index using the Accumulation of Deficits Model (cycles 1–3; *n* = 10,995) was developed; frailty was defined as having the presence of 25% or more indices, including symptoms, chronic conditions, and laboratory variables. Fried frailty (cycles 1–2; *n* = 7,353) included the presence of ≥3 criteria: exhaustion, physical inactivity, poor mobility, unintentional weight loss, and poor grip strength.

**Results:**

The prevalence of frailty was 8.6 and 6.6% with the Accumulation of Deficits and the Fried Model. Comparing the Fried vs. the Accumulation of Deficits Model, the prevalence of frailty was 5.3% vs. 1.8% in the 18–34 age group, 5.7% vs. 4.3% in the 35–49 age group, 6.9% vs. 11.6% in the 50–64 age group, and 7.8% vs. 20.2% in the 65+ age group. Some indices were higher in the younger age groups, including persistent cough, poor health compared to a year ago, and asthma for the accumulation of deficits model, and exhaustion, unintentional weight loss, and weak grip strength for the Fried model, compared to the older age groups.

**Conclusions:**

These data show that frailty is prevalent in younger adults, but varies depending on which frailty tool is used. Further research is needed to determine the health impact of frailty in younger adults.

**Electronic supplementary material:**

The online version of this article (doi:10.1186/s12877-017-0423-6) contains supplementary material, which is available to authorized users.

## Background

Frailty is linked to an increased risk of adverse health outcomes [1–5] and is characterized by reductions in physiologic reserve and a reduced ability to respond to stress [6]. Studies show that older adulthood is associated with a higher prevalence of frailty [4]. However, there is a paucity of evidence to determine if frailty can manifest at earlier ages [7]. Importantly, the scant evidence suggests that the relative risk of mortality in younger adults is associated more with frailty than age [7]. This poor health status even at a younger age could partially explain why many older adults thrive in their later years, while some younger adults fail to prosper. Indeed, aging is a heterogeneous process and chronologic age is not necessarily synonymous with an individual’s health status. Even so, there is little evidence to determine if there are differences in which frailty criteria are present in younger versus older adults.

While it is generally accepted that frailty increases with age, the identification of frailty is a challenge due to the development of multiple measurement tools and lack of consensus of which tool is most valid and feasible to implement in healthcare [8–10]. Two widely used frailty measures are the *Accumulation of Deficits model,* which uses a Frailty Index (FI) to characterize frailty as a state, and the *Fried model*, which describes frailty as a medical syndrome [11, 12]. The Fried model is distinct from disability and co-morbidity, while the FI is often inclusive of these conditions [13, 14]. These frailty tools are commonly used in older adults with few investigations in younger populations [4, 9]. At present, there is a lack of a large scale comparison between the Fried and FI exists in a Canadian population that spans the the adult lifespan.

Determination of the prevalence of frailty across adult age groups in the Canadian adult population is important for healthcare providers and policymakers. If the prevalence of frailty in younger adults is significant, implementation of screening at younger ages should be considered. Furthermore, understanding frailty is essential so as to provide targeted interventions towards specific frailty criteria, which are shown to be effective [15]. Indeed, the identification of which health issues are driving frailty in younger and older age groups can help design more focused interventions.

The purpose of this study was to compare the prevalence of frailty between the FI and Fried criteria in a large Canadian adult population. A second objective was to identify and compare which components of each frailty tool were present across the different age groups.

## Methods

### Study design

We used the cross-sectional Canadian Health Measures Survey (CHMS) from three data collection cycles [16].

### Study population

The CHMS is a representative cohort of the Canadian population 3–79 years old (*n* = 16,019). The present study included those 18–79 years old from the 3 CHMS cycles. A stratified random sample, where 11 age-gender groups with 500–600 units per group were used. Sixteen sampling sites, with five strata were used: Atlantic Canada, Quebec, Ontario, the Prairies, and British Columbia. Residents from the three territories, Aboriginal settlements, Crown lands, institutionalized individuals, remote regions, and full-time members of the Canadian Forces were excluded from CHMS recruitment. These excluded populations represent approximately 4% of the Canadian adult population. Data were collected from March 2007 to December 2013 across the three CHMS cycles. Household interviews were used to collect information on demographics, socioeconomic status, housing characteristics, and health status. Physical and laboratory measurements were collected in a mobile examination center.

### Ethics, consent and permissions

Ethical approval from our local research ethics board, Statistics Canada, Health Canada and the Public Health Agency of Canada was received for to access the CHMS datasets. Informed written consent was obtained from participants [16].

### Measurement and outcomes

#### Accumulation of deficits

The Accumulation of Deficits Model uses an FI to capture frailty based on the presence of signs, symptoms, laboratory values, chronic conditions, and disabilities. An FI was not developed in the three CHMS cycles; therefore, one was constructed using previously published guidelines [14]. Variables within the FI should increase with age, be associated with poor outcomes, cover a range of physiological systems, cannot be uncommon (<1%) or common (>80% by age 80), and more than 5% of variables cannot be missing for an individual. The FI is the ratio of health problems within the index. For example, someone with 6/23 deficits would score 0.26. A 23-item FI was created based on self-reported and laboratory-based variables (Additional file 1: Table S1). While it is recommended that an FI should have 30 variables, we chose a smaller FI due to a large number of missing laboratory variables. Previous studies have used fewer than 30 variables (as low as 15) and are associated with poor outocmes [17–19]. All variables were re-coded as a 0 (absence of deficit) or 1 (presence of deficit). A person was deemed frail in the present study if they scored 0.25/1 or higher on the FI [20].

### Fried frailty

The first two CHMS cycles had variables to capture frailty using the Fried model (*n* = 7,599). Fried frailty is identified based on the presence of three or more of the following: poor grip strength, slow gait speed, unintentional weight loss, exhaustion, and physical inactivity [11] Grip strength and unintentional weight loss in CHMS were measured in the same way as the original Fried frailty criteria. We modified the remaining variables using CHMS variables that were related to the original measure. Modification to the Fried methodology has been used previously and modified Fried frailty tools are similarly associated with health outcomes [21–23]. We used mobility issues as a proxy for gait speed, where participants self-reported the level of support needed to be mobile. Exhaustion was gauged by the question, “How often do you find it difficult to stay awake during your normal waking hours when you want to?” Physical activity levels were assessed with the CHMS Physical Activity Index, which codes participants based on level of activity. See Additional file 1: Table S2 for scoring of the Fried criteria.

### Statistical analysis

We evaluated differences in descriptive variables between CHMS cycles, age groups, and frailty classifications using the *t*-test, Mann-Whitney *U*-test, one-way analysis of variance, and the Kruskall Wallace test as appropriate for continuous variables and the Chi-square test or Fisher’s exact test for categorical variables. Sampling weights provided by Statistics Canada were used to account for survey representativeness and nonresponse [24]. Agreement between the FI and the Fried model was assessed using the Kappa statistic. To attenuate bias as a result of missing data and due to the relatively high probability of missing one of the 23 variables in the FI, we used multiple imputations. All analyses were carried out using SAS Version 9.4 (SAS Institute, Cary, North Carolina).

## Results

### Study cohort and participant characteristics

The response rates of participants after being approached to participate in CHMS cycles 1, 2 and 3 was 51.7, 55.5, and 51.7%, respectively. For the FI, 2091 had missing questionnaire data, and 2933 had missing laboratory values across the final included sample of 10,955 individuals. For the Fried model, 120 and 126 participants had missing self-report and handgrip strength data, respectively. This resulted in 7353 participants to estimate frailty based on the Fried model.

The participant characteristics across CHMS cycles can be viewed in Additional file 1: Table S3 and include the FI measures used in this study. Participants were middle-aged (~45 years old), 49% were female, and reported being in good health. Across the CHMS cycles, there were statistically significant differences for most of the laboratory values, although not necessarily clinically significant.

### Prevalence of frailty

#### Total prevalence

The prevalence of frailty was 7.6% using the FI (Table 1). Frail participants were approximately 14 years older than non-frail participants (*p* < 0.001) but males and females were equally likely to be frail. Significant differences between frail and non-frail participants were found for all FI variables except for resting heart rate and diastolic blood pressure.

**Table 1:** Comparison of health-deficits between frail and non-frail participants defined using the Frailty Index and Fried criteria

Variable	Accumulation of Deficits		Fried frailty	
	Not frail (*n* = 10,042)	Frail (*n* = 953)	Not frail (*n* = 6,871)	Frail (*n* = 482)
Age	43.94 (0.35)	57.98 (0.80)^c^	44.67 (0.42)	47.32 (1.13)^e^
Sex (% male)	4946 (49.3%)	497 (52.2%)	3506 (51.0%)	166 (34.5%)^e^
Diabetes^a^	317 (3.3%)	348 (36.6%)^c^	352 (5.1%)	52 (10.8%)^e^
Thyroid problem^a^	599 (5.9%)	206 (21.7%)^c^	443 (6.5%)	57 (11.8%)^d^
Cancer^a^	403 (4.0%)	181 (19.0%)^c^	304 (4.4%)	47 (9.8%)^e^
Stroke^a^	69 (0.7%)	44 (4.6%)^c^	58 (0.8%)	14 (2.9%)^d^
Heart disease^a^	253 (2.5%)	229 (24.0%)^c^	284 (4.1%)	46 (9.5%)^e^
Arthritis^a^	1266 (12.6%)	515 (54.1%)^c^	982 (14.3%)	160 (33.3%)^e^
Persistent cough^a^	1258 (12.5%)	354 (37.1%)^c^	954 (13.9%)	92 (19.2%)^d^
Known kidney dysfunction^a^	99 (1.00%)	94 (9.9%)^c^	109 (1.6%)	23 (4.7%)^e^
Poor health ^a^	788 (7.8%)	520 (54.6%)^c^	624 (9.1%)	172 (35.6%)^e^
Poor health compared to 1 year ago^a^	1113 (11.1%)	426 (44.7%)^c^	833 (12.1%)	139 (28.8%)^e^
COPD^a^	38 (0.4%)	72 (7.5%)^c^	47 (0.7%)	15 (3.1%)^e^
Asthma^a^	770 (7.7%)	245 (25.7%)^c^	602 (8.8%)	59 (12.3%)
Liver disease^a^	220 (2.2%)	92 (9.6%)^c^	199 (2.9%)	16 (3.3%)
Trouble sleeping^a^	4712 (47.0%)	708 (74.2%)^c^	3238 (47.1%)	304 (63.1%)^e^
Resting HR (beats/min)^a^	68.21 (0.30)	68.97 (0.72)	67.61 (0.29)	69.35 (0.61)^e^
Systolic BP (mmHg)^a^	111.41 (0.40)	120.80 (1.46)^c^	112.43 (0.69)	113.32 (1.37)
Diastolic BP (mmHg)^a^	71.33 (0.21)	71.96 (0.75)	71.77 (0.41)	70.54 (1.02)^e^
eGFR (ml/min)	106.05 (0.47)	91.43 (1.21)^c^	103.90 (0.61)	104.65 (1.66)
Albumin (g/L)^a^	44.79 (0.17)	44.16 (0.29)^c^	45.26 (0.15)	43.49 (0.43)^e^
Haemoglobin (g/L)	142.51 (0.46)	140.04 (0.96)^c^	143.09 (0.46)	138.84 (1.07)^e^
Calcium (mmol/L)	2.41 (0.00)	2.43 (0.01)^c^	2.41 (0.00)	2.39 (0.01)^e^
Phosphate (mmol/L)	1.25 (0.01)	1.28 (0.02)^c^	1.23 (0.00)	1.23 (0.01)^e^
RBC count (10-12/L)^a^	4.66 (0.01)	4.60 (0.05)^b^	4.67 (0.02)	4.54 (0.03)^e^
RBC distribution width (%)^a^	12.69 (0.04)	13.38 (0.09)^c^	12.51 (0.03)	12.92 (0.09)^d^
Aspartate Aminotransferase (U/L)^a^	27.94 (0.37)	33.80 (0.90)^c^	28.31 (0.29)	26.92 (0.72)^d^
HbA1_C_ (%)^a^	5.53% (0.02%)	6.45% (0.09%)^c^	5.67% (0.04%)	5.89% (0.07%)^e^
Plasma glucose (mmol/L)^a^	4.99 (0.02)	6.38 (0.15)^c^	5.08 (0.03)	5.43 (0.12)^e^

^a^indicates variables included in the Frailty Index. ^b^ *P* < 0.05 for Non-frail vs. frail for frailty index. ^c^ *P* < 0.001 for non-frail vs. frail for frailty index. ^d^ *P* < 0.05 for Non-frail vs. frail for frailty index. ^e^ *P* < 0.001 for non-frail vs. frail for Fried criteria Categorical variables are displayed as frequency (%) and continuous variables are shown as mean (standard deviation). Frailty was defined as having 3–5 of the Fried criteria. *COPD* chronic obstructive pulmonary disease, *HR* heart rate, *BP* blood pressure, *eGFR* estimated glomerular filtration rate, *RBC* red blood cell

The prevalence of frailty with the Fried criteria was 6.2%. On average frail participants were 3 years older than their non-frail peers (*p* = 0.006). Females were more likely to be frail compared to males (*p* < 0.001). Among the health-related variables, significant differences between frail versus non-frail individuals were found for the majority of values except asthma, liver disease, systolic blood pressure, estimated glomerular filtration rate, and phosphate (Table 1). A comparison of the frequency of individual Fried frailty criteria was made between frail and non-frail participants (Table 2). The most prevalent frailty criteria were exhaustion and physical inactivity.

**Table 2:** Comparison of the individual Fried frailty criteria among frail versus non-frail participants defined using the Fried frailty criteria

Variable	Frequency of Fried criteria based on Cycles 1 and 2 (*n* = 7,353)
Exhaustion (% yes)	2269 (30.9%)
Physically inactive	3917 (53.3%)
Mobility function (gait speed proxy)	197 (2.7%)
Unintentional weight loss	1361 (18.5%)
Weak grip strength	409 (5.6%)
Total number of Fried criteria	
0	2038 (27.7%)
1	3127 (42.5%)
2	1734 (23.6%)
3	369 (5.0%)
4+	112 (1.7%)

Variables are displayed as frequency (%)

### Frailty prevalence across age groups

**Fig. 1:**
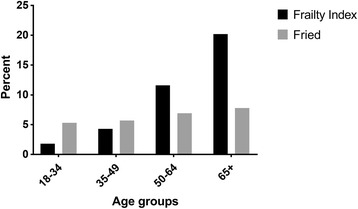
Prevalence of frailty across age categories and frailty definition

A comparison of frailty prevalence across age categories and between frailty measures within the first two CHMS cycles can be viewed in Fig. 1. The number of participants deemed frail with the FI was 1.8, 4.3, 11.6, and 20.2% in the 18–34, 35–49, 50–64, and 65+ age categories, respectively. With the Fried model, 5.3%, 5.7%, 6.9%, 7.8% were frail in the 18–34, 35–49, 50–64, and the 65+ age categories, respectively. The agreement between frailty measures for the entire cohort was 0.132 (95% CI, 0.131–0.133). Across age groups, the agreement between the two frailty measures was −0.017 (95% CI, −0.018–0.017) for the 18–34 group, 0.077 (95% CI, 0.076–0.078) for the 35–49 group, 0.165 (95% CI, 0.164–0.167) for the 50–64 group, and 0.197 (95% CI, 0.196–0.199) for the 65+ group.

In individuals who were deemed as frail using the FI, the proportion of those with or without each component of the FI were compared (Table 3). Data was censored for stroke, heart disease, and chronic obstructive pulmonary disease due to the low prevalence in the 18–49 age group. The older age groups tended to have a higher prevalence of individual frailty variables. There were no differences for gender, thyroid problems, kidney dysfunction, poor self-perceived health, liver disease, and trouble sleeping across age groups. There was a significantly higher prevalence of persistent cough, poor self-perceived health compared to 1 year ago, and asthma in the younger compared to the older age groups.

**Table 3:** Comparison of health-deficits across age categories among frail only participants defined using the frailty index

Variable	18–49 years (*n* = 574)	50–64 years (*n* = 254)	65+ years (*n* = 125)	Chi-Square or ANOVA F-Test *P*-value
Age	37.90 (1.04)	57.90 (0.42)	72.07 (0.26)	<0.001
Sex (% male)	327 (57.0%)	135 (53.2%)	60 (47.7%)	0.399
Diabetes	101 (17.5%)	104 (41.1%)	56 (44.5%)	<0.001
Thyroid problem	119 (20.7%)	53 (20.7%)	29 (23.5%)	0.841
Cancer	41 (7.2%)	61 (24.00%)	27 (21.3%)	<0.001
Stroke^a^	-	-	-	-
Heart disease^a^	-	-	-	-
Arthritis	136 (23.8%)	150 (59.0%)	87 (69.4%)	<0.001
Persistent cough	281 (48.9%)	85 (33.4%)	42 (33.3%)	0.010
Known kidney dysfunction	44 (7.7%)	23 (9.1%)	15 (12.3%)	0.442
Poor health	333 (58.00%)	143 (56.3%)	63 (50.1%)	0.581
Poor health compared to 1 year ago	320 (55.7%)	114 (44.8%)	46 (36.9%)	0.033
COPD^a^	-	-	-	-
Asthma	244 (42.6%)	62 (24.4%)	19 (15.4%)	<0.001
Liver disease	51 (8.9%)	29 (11.0%)	10 (7.9%)	0.549
Trouble sleeping	459 (79.9%)	194 (76.3%)	85 (67.9%)	0.213
Resting HR (beats/min)	70.35 (1.17)	71.08 (1.25)	65.55 (0.66)	<0.001
Systolic BP (mmHg)	114.53 (2.76)	120.11 (1.90)	125.97 (1.27)	<0.001
Diastolic BP (mmHg)	74.52 (1.87)	73.42 (1.04)	68.48 (0.60)	<0.001
eGFR (ml/min)	108.62 (2.31)	94.16 (1.30)	76.13 (2.15)	<0.001
Albumin (g/L)	45.26 (0.83)	44.20 (0.35)	43.33 (0.29)	<0.001
Haemoglobin (g/L)	142.82 (2.44)	142.02 (0.95)	135.73 (1.70)	<0.001
Calcium (mmol/L)	2.44 (0.02)	2.43 (0.01)	2.42 (0.01)	0.023
Phosphate (mmol/L)	1.31 (0.03)	1.30 (0.02)	1.24 (0.01)	<0.001
RBC count (10–12/L)	4.71 (0.13)	4.69 (0.08)	4.43 (0.05)	<0.001
RBC distribution width (%)	13.33 (0.19)	13.27 (0.18)	13.54 (0.09)	0.043
Aspartate Aminotransferase (U/L)	37.09 (2.52)	34.68 (1.67)	30.47 (0.87)	<0.001
HbA1_C_ (%)	6.12% (0.15%)	6.6% (0.15%)	6.5% (0.08%)	<0.001
Plasma glucose (mmol/L)	5.97 (0.32)	6.52 (0.25)	6.50 (0.19)	0.037

^a^ *n*-value too low in the 18–49 category and could not report on numbers based on Canadian Health Measures ethics. Categorical variables are displayed as frequency (%) and continuous variables are shown as mean (standard deviation). Frailty was defined as having 3–5 of the Fried criteria. *COPD* chronic obstructive pulmonary disease, *HR* heart rate, *BP* blood pressure, *eGFR* estimated glomerular filtration rate, *RBC* red blood cell

The individual Fried frailty criteria among frail participants across age categories were compared (Table 4). Data was not released from the Research Data Center for physical activity and were censored due to almost 100% of participants being classified as inactive by the Physical Activity Index. The prevalence of poor mobility based on the Fried criteria increased in the older age groups. The prevalence of exhaustion, unintentional weight loss, and weak grip strength were higher in the younger age groups compared to the 65+ group.

**Table 4:** Comparison of individual Fried frailty criteria across age categories among frail only participants defined using the Fried frailty criteria

Variable	18–49 years (*n* = 263)	50–64 years (*n* = 142)	65+ years (*n* = 77)	Chi-Square *P*-value
Exhaustion (% yes)	139 (52.9%)	34 (24.0%)	8 (11.0%)	<0.001
Physical activity (% inactive)^a^	-	-	-	<0.001
Mobility function (gait speed proxy, % mobility impairment)	13 (4.9%)	13 (9.3%)	7 (9.7%)	<0.001
Unintentional weight loss (% yes)	127 (48.1%)	29 (20.1%)	5 (7.1%)	<0.001
Weak grip strength (% yes)	33 (12.6%)	17 (12.2%)	9 (11.9%)	<0.001

^a^Almost all participants scored positive on the physically inactive category and thus did not report percentages based on Canadian Health Measures ethics. Categorical variables are displayed as frequency (%) and continuous variables are shown as mean (standard deviation). Frailty was defined as having 3–5 of the Fried criteria

## Discussion

In this representative study of Canadian adults 18–79 years old, the overall prevalence of frailty was 6.6% using a modified Fried model and 7.6% using a FI. Frailty was more common in older individuals, but the prevalence in each age category differed depending on the model used [11, 12]. When the frail only participants were examined, the prevalence of individual frailty criteria differed between the younger and older age groups (Tables 3 and 4). These findings suggest that frailty can be prevalent at any age and may present differently in younger versus older adults.

The findings of previous studies using older cohorts demonstrate that the prevalence of frailty is higher with the FI versus the Fried criteria are further supported by the present study [4, 25–27]. Similarly, in a systematic review, the prevalence of frailty was 12% using the Fried model and 24% using the Accumulation of Deficits Model in community-dwelling adults 65 years or older [4]. In addition, our findings are similar to those in the Canadian National Population Health Survey which showed an increase in the prevalence of frailty from 2 to 22% in those aged younger than 30 and 65 years or older, using an FI [7]. Our findings indicate that frailty generally was not significantly different between males and females (except for the Fried frailty model amongst the entire cohort), which contrast previous studies [28–31]. It is possible that included health deficits, which captured mostly chronic conditions and laboratory values, may underestimate frailty in women. For example, women tend to have higher rates of depression and anxiety [32], which were excluded in the FI in the present study. Furthermore, some of the chronic conditions within the FI tend to develop later in life in women (e.g., cardiovascular diseases) – given our relatively young sample, this could be another possible explanation for the mostly non-significant findings in frailty prevalence between men and women.

The agreement between the two frailty measures in our study was low, which is common when comparing frailty scales. In fact, eight frailty scales were compared in the Survey of Health, Ageing and Retirement in Europe cohort, which included the FI and Fried criteria, showed that all the scales categorized less than 3% of participants as frail [27]. Although there was a higher agreement between the FI and the Fried criteria in that study compared to the present study, our inclusion of a younger cohort compared to the aforementioned study could account for this difference. There is also a possibility that the two frailty measures used in this study could be capturing groups of individuals who may or may not be vulnerable to poor health outcomes, which needs to be explored in further studies.

The evidence generated from our study indicates that it is feasible to measure frailty in younger adults. Further research is needed to explore the feasibility and value of frailty screening for adults of all ages [33]. However, given the paucity of evidence describing the prevalence and health impact of frailty in younger adults, it is still too early to recommend frailty screening in this age group. Possible benefits to frailty screening, at least in the older population, is providing additional risk assessment for those requiring invasive procedures. For example, frailty is shown to increase one’s risk for postoperative cardiac-surgical outcomes [3]; identifying someone who is frail could lead to more conservative approaches (e.g., trans-catheter aortic valve replacement) that could help maximize a frail older adult’s quality of life. On the other hand, frailty screening could lead to incorrectly identifying someone as frail who is not and might result in withholding beneficial treatments in favor of more conservative approaches.

Considering that our data shows that younger adults (18+) can be frail, it warrants future investigation to provide clarity as to whether frailty similarly impacts poor health outcomes in the young versus the old. More specifically, further research is needed to determine the prevalence of frailty, its potential health impact in the young, and whether frailty in younger age groups is associated with the use of more healthcare resources, as compared to their non-frail peers. While both the FI and Fried criteria are predictive of mortality in older adults [4], our study warrants that studies should be conducted to determine the health impact of frailty in younger age cohorts, and which tool could be used to most accurately assess their future health risk. Evidence suggests that the FI has better prognostic ability for predicting mortality in older adults than the Fried criteria in the short to medium term [27, 34], but to our knowledge, there has been no comparison of their prognosis in younger adults. This nascent evidence must be strengthened, with further investigation needed in younger population-based cohorts.

In order to determine the health impact of frailty in younger populations, proponents of research in frailty should consider linking CHMS data with administrative health databases. Furthermore, while the FI is suggested to provide better prognostication than the Fried model in older adults [34], it is unclear if the FI or Fried criteria (or other frailty tools) have better predictive validity for determining adverse outcomes in younger adults. Our data also show that the Fried model estimated a higher frailty prevalence in the 18–34 age group (5.3%) compared to the FI (1.8%), which needs further exploration. Among the individual Fried frailty criteria, it is interesting that approximately half of the younger cohorts scored positive on exhaustion and unintentional weight loss which suggests that targets to prevent or treat frailty might differ compared to the older age groups with an increasing prevalence of impaired mobility. Collectively, the evidence base of frailty in younger age groups must be strengthened.

### Strengths and limitations

The data presented are from a large representative sample of over 95% of the adult Canadian population who are 18–79 years old. In addition, this study investigated the prevalence and factors associated with frailty in younger adults, a population that has previously not been studied in detail. Our data suggest that frailty is not synonymous with chronologic age, and younger adults who are already frail could place a significant burden on the healthcare system. This study investigated the prevalence of frailty using two of the multiple frailty instruments available [8–10]. The two frailty tools used in this study are the most widely used in previous studies.

There are limitations with this study. Due to a significant number of missing laboratory variables, the decision was made to exclude a number of variables from the FI. Thus, the number of FI variables in the present study was reduced, potentially affecting criterion and external validity. The individual FI variables in this study also mostly cover a range of chronic conditions rather than a range of other domains, including cognitive, psychological, or functional domains, which could potentially impact the prevalence of frailty in our study. This was in part due to maximizing our sample size. Despite lacking those domains in the present study, the prevalence of frailty across age groups matched well with a previous Canadian study in adults across the lifespan [7]. Also, we used multiple imputation to maximize the sample size, which may have skewed our results. We also modified the Fried criteria based on the variables available in the CHMS cohort. Many of the younger age groups screened positive for exhaustion (52.9%), physical inactivity (almost all), and unintentional weight loss (48.1%) with the Fried model. Therefore, it is possible that the high rates of these modified Fried criteria could have overestimated frailty status in the younger age groups. Much like the differences in prevalence estimates when comparing the FI and Fried frailty model, modifications to the Fried frailty model can significantly impact the estimated prevalence of frailty (13–28%) [35]. However, previous studies show that a modified Fried criteria is associated with adverse events [26, 27, 36]. Lastly, we dichotomized participants into frail versus not frail, where some important information might be lost, such as the severity of frailty.

## Conclusion

Our study found that frailty is prevalent at any adult age. In addition, the prevalence of frailty differed between the FI and Fried models and the prevalence was higher in the younger age categories using the Fried model. As well, the prevalence of components of frailty was different in younger age groups as compared with older age groups. Future studies should investigate the factors that drive frailty in younger adults, the utility of screening for frailty in the younger adult population, and whether interventions can improve frail status and associated outcomes across the adult lifespan.

## Additional file

Additional file 1: Table S1. Components of the frailty index and how they are scored. **Table S2.** Components of the Fried frailty components and how they are scored. **Table S3.** Comparison of health-deficits across Canadian Health Measures Cycles. (DOCX 17 kb)

## Abbreviations

CHMS: Canadian Health Measures Survey
FI: Frailty Index
