# Supplementary material for: Prevalence of frailty in Canadians 18–79 years old in the Canadian Health Measures Survey
Source: BMC Geriatr. 2017 Jan 21;17:28. doi: 10.1186/s12877-017-0423-6 (PMC5251297; doi:10.1186/s12877-017-0423-6)
Supplement: Additional file 1: Table S1. — Components of the frailty index and how they are scored. Table S2. Components of the Fried frailty components and how they are scored. Table S3. Comparison of health-deficits across Canadian Health Measures Cycles. (DOCX 17 kb) [file 12877_2017_423_MOESM1_ESM.docx]

**Additional file 1**

**Table S1.** Components of the frailty index and how they are scored.

| **Deficit:** | **Coding (0=no deficit, 1= deficit)** |
| --- | --- |
|  |  |
| Diabetes | 0= no; 1= yes |
| Thyroid problems |  |
| Cancer |  |
| Stroke |  |
| Heart disease |  |
| Arthritis |  |
| Liver disease |  |
| Kidney disease |  |
| Chronic obstructive pulmonary disease |  |
| Asthma |  |
| Persistent cough |  |
| Poor self-perceived health |  |
| Poor self-perceived health compared to one year ago |  |
| Sleeping problems |  |
| Heart rate (bpm) | 0= 60-100; 1= <60 or >100 |
| Systolic blood pressure (mmHg) | 0= <140; 1= >140 |
| Diastolic blood pressure (mmHg) | 0= 60-100; 1= 60 or >100 |
| Albumin (g/L) | 0= 32-44; 1= <32 or >44 |
| Plasma glucose (mmol/L) | 0= 3.9-6.1; 1= <3.9 or >6.1 |
| Red blood cell count (10-12/L) | 0= 3.93-5.69; 1= <3.93 or >5.69 |
| Red blood cell width (%) | 0= 11.5-14.5; 1= <11.5 or >14.5 |
| Aspartate aminotransferase (U/L) | 0= 8-33; 1= <8 or >33 |
| HbA1_c_ (%) | 0= 4.5-6.4; 1= <4.5 or >6.4 |

**Table S2.** Components of the Fried frailty components and how they are scored.

| **Frailty component** | **Coding (0= absence of criteria, 1= presence of criteria)** |
| --- | --- |
| Unintentional weight loss | 0= weight change intentional or not applicable; 1= weight change unintentional |
| Exhaustion | 0= never or rarely; 0.5= sometimes; 1= most of the time or all of the time |
| Mobility problems | 0= no mobility problems; 0.25= some problem – no aid required; 0.5= requires mechanical support; 0.75= requires wheelchair; 1= requires help from people or cannot walk |
| Grip strength | Sex and BMI adjusted based on the original Fried publication (below values indicate that they would score positive (i.e., a “1”).  Men cut-off values:  BMI ≤24 – grip strength ≤29kg  BMI 24.1-28 – grip strength ≤30kg  BMI >28 – grip strength ≤32kg  Female cut-off values:  BMI ≤23 – grip strength ≤17kg  BMI 23.1-26 – grip strength ≤17.3kg  BMI 26.1-29 – grip strength ≤18kg  BMI >29 – grip strength ≤21kg |
| Physical activity | 0=Physical Activity Index score where participants are either Moderately Active or Active. 1= Physical Activity Index score where participants are Inactive. |

**Table S3.** Comparison of health-deficits across Canadian Health Measures Cycles

| **Variable** | **Cycle 1**  **(n= 3726)** | **Cycle 2**  **(n= 3873)** | **Cycle 3**  **(n= 3396)** | **Chi-Square or ANOVA F-Test**  **P-value** |
| --- | --- | --- | --- | --- |
| Age | 44.58 (0.66) | 45.00 (0.56) | 45.52 (0.63) | 0.0805 |
| Sex (% male) | 1837 (49.31%) | 1916 (49.48%) | 1686 (49.64%) | 0.9843 |
| Diabetes^a^ | 175 (4.70%) | 236 (6.10%) | 211 (6.20%) | 0.1819 |
| Thyroid problem^a^ | 294 (7.90%) | 236 (6.10%) | 251 (7.40%) | 0.2642 |
| Cancer^a^ | 171 (4.60%) | 182 (4.70%) | 204 (6.00%) | 0.2392 |
| Stroke^a^ | 34 (0.90%) | 43 (1.10%) | 32 (0.95%) | 0.8188 |
| Heart disease^a^ | 190 (5.10%) | 160 (4.12%) | 113 (3.32%) | 0.0815 |
| Arthritis^a^ | 570 (15.30%) | 616 (15.90%) | 543 (16.00%) | 0.9149 |
| Persistent cough^a^ | 543 (14.56%) | 534 (13.79%) | 503 (14.82%) | 0.8423 |
| Known kidney dysfunction^a^ | 60 (1.60%) | 88 (2.26%) | 39 (1.15%) | 0.0112 |
| Poor self-perceived health^a^ | 408 (10.96%) | 443 (11.44%) | 399 (11.74%) | 0.8158 |
| Poor self-perceived health compared to 1 year ago^a^ | 503 (13.49%) | 513 (13.25%) | 481 (14.16%) | 0.8083 |
| COPD^a^ | 31 (0.82%) | 34 (0.88%) | 36 (1.07%) | 0.7567 |
| Asthma^a^ | 284 (7.63%) | 390 (10.07%) | 318 (9.35%) | 0.2758 |
| Liver disease^a^ | 130 (3.48%) | 89 (2.31%) | 85 (2.52%) | 0.0977 |
| Trouble sleeping^a^ | 1761 (47.27%) | 1890 (48.80%) | 1733 (51.05%) | 0.1746 |
| Resting HR (beats/min) ^a^ | 67.88 (0.42) | 67.84 (0.37) | 69.05 (0.52) | <0.0001 |
| Systolic BP (mmHg) ^a^ | 112.71 (0.80) | 112.11 (0.78) | 111.58 (0.49) | 0.0075 |
| Diastolic BP (mmHg) ^a^ | 71.97 (0.47) | 71.31 (0.46) | 70.87 (0.35) | <0.0001 |
| eGFR (ml/min) | 105.76 (0.96) | 102.99 (0.56) | 106.07 (0.99) | <0.0001 |
| Albumin (g/L) ^a^ | 46.23 (0.13) | 44.11 (0.19) | 43.96 (0.31) | <0.0001 |
| Haemoglobin (g/L) | 142.13 (0.53) | 142.94 (0.63) | 141.90 (0.76) | 0.0025 |
| Calcium (mmol/L) | 2.41 (0.01) | 2.41 (0.00) | 2.41 (0.01) | <0.0001 |
| Phosphate (mmol/L) | 1.23 (0.01) | 1.23 (0.01) | 1.30 (0.01) | <0.0001 |
| RBC count (10-12/L) ^a^ | 4.68 (0.02) | 4.64 (0.02) | 4.66 (0.02) | <0.0001 |
| RBC distribution width (%)^a^ | 12.52% (0.04%) | 12.57% (0.04%) | 13.12% (0.11%) | <0.0001 |
| Aspartate Aminotransferase (U/L) ^a^ | 27.83 (0.35) | 28.45 (0.42) | 28.85 (0.70) | 0.0021 |
| HbA1_C_ (%)^a^ | 5.62% (0.06%) | 5.73% (0.07%) | 5.45% (0.04%) | <0.0001 |
| Plasma glucose (mmol/L) ^a^ | 5.03 (0.02) | 5.16 (0.05) | 5.08 (0.06) | <0.0001 |
| ^a^indicates variables included in the Frailty Index. Categorical variables are displayed as frequency (%) and continuous variables are shown as mean (standard deviation). COPD, chronic obstructive pulmonary disease. HR, heart rate. BP, blood pressure. eGFR, estimated glomerular filtration rate. RBC, red blood cell. | | | | |
